# Effect of Earlier Door-to-CT and Door-to-Bleeding Control in Severe Blunt Trauma: A Retrospective Cohort Study

**DOI:** 10.3390/jcm10071522

**Published:** 2021-04-06

**Authors:** Shuhei Murao, Kazuma Yamakawa, Daijiro Kabata, Takahiro Kinoshita, Yutaka Umemura, Ayumi Shintani, Satoshi Fujimi

**Affiliations:** 1 Division of Trauma and Surgical Critical Care, Osaka General Medical Center, Osaka 558-8558, Japan; Japanshmu20268271@gmail.com (S.M.); plum0022@yahoo.co.jp (Y.U.); sfujimi40@nifty.com (S.F.); 2 Department of Emergency Medicine, Osaka Medical College, Takatsuki 569-8686, Japan; 3 Department of Medical Statistics, Osaka City University Graduate School of Medicine, Osaka 545-8586, Japan; kabata.daijiro@med.osaka-cu.ac.jp (D.K.); shintani.ayumi@med.osaka-cu.ac.jp (A.S.); 4 Department of Traumatology and Acute Critical Medicine, Osaka University Graduate School of Medicine, Suita 565-0871, Japan; ogmc.dmat.t.kinoshita@gmail.com

**Keywords:** angioembolization, emergency surgery, hemorrhagic shock, interventional radiology, laparotomy, thoracotomy, whole-body CT

## Abstract

Blunt trauma is a potentially life-threatening injury that requires prompt diagnostic examination and therapeutic intervention. Nevertheless, how impactful a rapid response time is on mortality or functional outcomes has not been well-investigated. This study aimed to evaluate effects of earlier door-to-computed tomography time (D2CT) and door-to-bleeding control time (D2BC) on clinical outcomes in severe blunt trauma. This was a single-center, retrospective cohort study of patients with severe blunt trauma (Injury Severity Score > 16). To assess the effect of earlier D2CT and D2BC on clinical outcomes, we conducted multivariable regression analyses with a consideration for nonlinear associations. Among 671 patients with severe blunt trauma who underwent CT scanning, 163 patients received an emergency bleeding control procedure. The median D2CT and D2BC were 19 min and 57 min, respectively. In a Cox proportional hazard regression model, earlier D2CT was not associated with improved 28-day mortality (*p* = 0.30), but it was significantly associated with decreased mortality from exsanguination (*p* = 0.003). Earlier D2BC was significantly associated with improved 28-day mortality (*p* = 0.026). In conclusion, earlier time to a hemostatic procedure was independently associated with decreased mortality. Meanwhile, time benefits of earlier CT examination were not observed for overall survival but were observed for decreased mortality from exsanguination.

## 1. Introduction

Traumatic injury is an important public health issue causing more than five million deaths annually, and it is the major cause of death among young adults [1]. Uncontrolled hemorrhage is the leading cause of potentially preventable death [2,3,4]. Approximately 20–40% of trauma deaths occurring after hospital arrival involve massive hemorrhage from truncal injury and are potentially preventable with early bleeding control procedures and improved resuscitation techniques [5]. Sufficient therapy within the first hour after injury significantly increases outcome in these patients. Thus, identifying and quickly controlling hemorrhage and initiating resuscitation are pivotal steps in assessing and managing trauma patients.

To accomplish rapid bleeding control in expectation of reducing preventable death, the diagnostic work-up of severely injured patients is a matter of ongoing development. Computed tomography (CT) is an advanced imaging modality that offers high sensitivity in identifying life-threatening injuries [6,7,8]. Whole-body CT scanning has become technically feasible after the introduction of rapid and accurate multi-detector CT scanners, and it is commonly used in trauma centers as a single-pass primary assessment [9,10,11]. A recent study reported that earlier time to diagnosis for life-threatening injuries was attained with whole-body CT scanning versus a standard diagnostic work-up [12]. Furthermore, several institutions have introduced CT scanners in their trauma resuscitation rooms to eliminate patient transportation time, which have contributed to decrease the time of CT examination, decreased time to control bleeding, and decreased death from exsanguination [13,14,15,16,17,18,19]. However, little evidence is available with regard to the beneficial effects of earlier times to CT examination and hemostatic treatment in trauma patients.

Thus, the aim of this study was to evaluate whether earlier door-to-CT time (D2CT) and door-to-bleeding control time (D2BC) have a beneficial impact on survival in patients with severe blunt trauma. To identify these associations, we estimated the nonlinear effect of D2CT and D2BC on each clinical outcome using multivariable regression models with adjustment for demographic and clinical covariates.

## 2. Materials and Methods

### 2.1. Study Design

This was a single-center, retrospective cohort study conducted at a tertiary hospital in Osaka, Japan. The study followed the Declaration of Helsinki and was approved by the Institutional Review Board at Osaka General Medical Center (approval no. 30-S11-002). The board waived the need for informed consent as this was a retrospective study.

### 2.2. Patient Population

Records were retrospectively reviewed from patients with severe blunt trauma (Injury Severity Score [ISS] ≥ 16) treated between August 2007 and July 2015. We included patients who underwent whole-body CT scanning within 90 min of emergency room arrival. Exclusion criteria included patients who were transferred from other hospitals and not treated in the trauma resuscitation room, patients with traumatic cardiopulmonary arrest on arrival, pediatric patients younger than 15 years, patients who were transferred to other hospitals within 24 h after admission, and pregnant women. Patients who underwent CT within two min after emergency room arrival and patients who received emergency surgery before CT scanning were also excluded in an effort to include patients who would receive the most benefits from CT scanning. Furthermore, in our assessment of the effects of D2BC, the dataset included subjects who received any bleeding control treatment.

### 2.3. Trauma Management Policy

At the halfway point of the study period (August 2011), our hospital installed an angio-CT system in a trauma resuscitation room. As this system enabled us to conduct CT examinations immediately after patient arrival without transfer to a CT room, the indication for CT scanning in patients with potentially life-threatening injury changed at this time point. However, the fundamental concepts of trauma management were based on ATLS methods throughout the entire study period [20].

When patients arrived at the trauma resuscitation room, a trauma team completed the primary assessment, and an attending physician decided whether to perform CT scanning. Hemodynamically stable patients or patients who rapidly responded to the initial fluid resuscitation were assumed to be able to tolerate CT scanning in the first study period in which the CT scanner was on the same floor as, but not in, the trauma resuscitation room. In the latter period, a systolic blood pressure (BP) of 70 mmHg was regarded as the threshold for conducting CT scanning or immediate resuscitative procedures including resuscitative thoracotomy and resuscitative endovascular balloon occlusion of the aorta (REBOA). All patients included in this study underwent CT examinations performed using our institutional trauma CT protocol. Images were acquired by using multi-slice CT scanner (Aquilion CX, TSX-101A; Toshiba Medical System Corp., Tochigi, Japan). CT protocol in blunt trauma patients includes an unenhanced CT head, followed by an arterial and venous phases for chest and abdominopelvic CT. Attending emergency physicians evaluated each patient’s CT scan. If certain abnormalities were clearly identified in the primary survey or CT scanning was difficult due to hemodynamic instability, emergency bleeding control surgery was performed in the trauma resuscitation room.

Another feature of our institution is that all trauma surgeons are trained to perform both general surgery and interventional radiology. Therefore, we can start not only emergency laparotomy and thoracotomy but also interventional radiology procedures including REBOA and angioembolization without significant time delay after patient arrival.

### 2.4. Data Extraction

The emergency department variables (Glasgow Coma Scale, systolic BP, heart rate (HR), shock index, body temperature (BT), respiratory rate, pH, base excess, lactate value, hemoglobin (Hb), prothrombin time-international normalized ratio (PT-INR), and activated partial thromboplastin time) were recorded as the initial set of vital signs and laboratory tests. The existence of trauma’s deathly triad of hyper hyperthermia hypothermia (BT < 35 °C), acidosis (pH < 7.2), and coagulopathy (PT-INR > 1.5) were evaluated during the first 24 h. The Abbreviated Injury Scale (AIS) of each body region was recorded and the ISS, Revised Trauma Score (RTS) and probability of survival were calculated using the Trauma and Injury Severity Score (TRISS) method. We recorded emergency bleeding control procedures under the following groups: direct bleeding control surgery (thoracotomy, laparotomy, preperitoneal pelvic packing, and others) and interventional radiology (chest, abdomen, pelvis, and others). Bleeding control treatment included both direct bleeding control surgery and interventional radiology. The D2CT (time from emergency room arrival to initiation of CT) and the D2BC (time from emergency room arrival to initiation of bleeding control procedure) were recorded. The time course of trauma workflow including D2CT and D2BC was documented by nurses and also recorded by video.

The primary outcome measure for analysis was 28-day mortality. The secondary outcome measures were 24-h mortality, the Oxford Handicap Scale (OHS), and the cause of death. The OHS was evaluated at 28 days or the day of hospital discharge, whichever occurred first, with handicap categories of independent (Grade 0 to 2), dependent (Grade 3 to 5), or death (Grade 6). The cause of death was categorized as exsanguination, traumatic brain injury (TBI), respiratory failure, sepsis, multiple organ dysfunction syndrome (MODS), and others.

### 2.5. Statistical Analysis

Descriptive statistics were calculated as medians (interquartile range) or proportions (numbers), as appropriate. To examine the effect of D2CT on 28-day mortality, we performed a multivariable Cox proportional hazard regression model with adjustment for the following covariates described in Table S1: age, sex, mechanism of injury, ISS, RTS, presence of deadly coagulopathy, presence of acidosis, presence of hypothermia, presence of bleeding control procedure, lactate, Sequential Organ Failure Assessment (SOFA) score on day 1, HR, BT, pH, Hb, and PT-INR. We assessed the effect of D2CT on the occurrence of 24-h mortality using a multivariable logistic regression model with adjustment for the same covariates described above except for SOFA score on day 1, HR, BT, pH, Hb, and PT-INR to avoid overfitting. The effect on OHS was also assessed by a multivariable proportional-odd logistic regression model with adjustment for the covariates considered in the Cox proportional hazard regression model. In this model, the fatal cases were categorized as Grade 6 of the OHS. Moreover, we evaluated the effect of D2CT on 24-h mortality from exsanguination in patients requiring a bleeding control procedure with adjustment for age, sex, mechanism of injury, ISS, and RTS. Finally, we assessed the effect of D2CT on 28-day mortality and 24-h mortality from TBI in patients requiring intracranial surgery adjusted for age, sex, mechanism of injury, ISS, RTS, presence of deadly coagulopathy, and presence of acidosis. For assessment of non-linear associations between D2CT and each outcome variable, we applied the restricted-cubic-spline method to D2CT in all models. All missing values were imputed by a multiple imputation method using the “aregImpute” function in the “rms” package [21]. Then, we conducted similar analyses to estimate the effect of D2BC on each outcome variable in the cohort with bleeding control. In these models, the presence of a bleeding control procedure was removed from the adjustment covariates. In the logistic regression model assessing the effect on 24-h mortality, we considered only age, sex, mechanism of injury, ISS, and RTS to avoid overfitting. Non-linearity and missing-value imputations were conducted similarly to the regression models assessing the effect of D2CT.

All statistical inference and hypothesis testing was conducted with a two-sided 5% significance level using R software version 3.6.1 (https://cran.r-project.org/ Accessed 27 September 2019]).

## 3. Results

### 3.1. Baseline Characteristics

**Figure 1:**
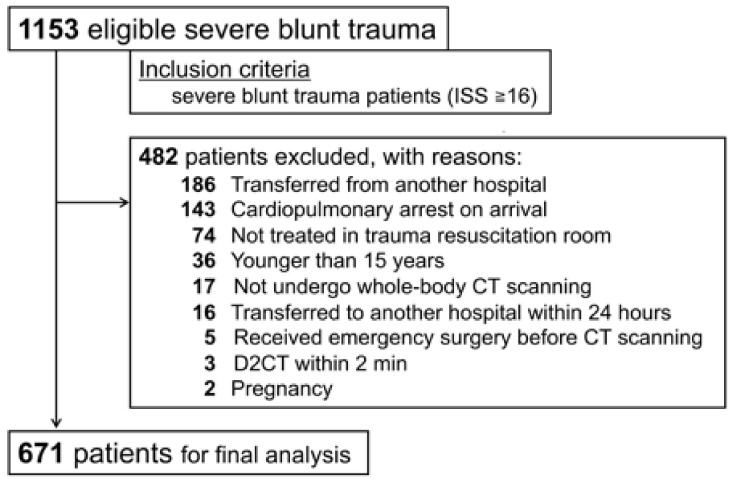
Patient flow diagram. ISS, Injury Severity Score; CT, computed tomography and D2CT, door-to-computed tomography time.

Of 1153 potentially eligible patients treated during the eight-year study period, 671 patients with severe blunt trauma who underwent CT scanning were included in the analysis (Figure 1). Table 1 summarizes the patients’ baseline characteristics. The median age of the included patients was 51 (35 to 65) years, and 463 patients (69.0%) were male. The median ISS was 26 (21–35). The mechanisms of injury were mostly motor vehicle injury (55.1%) and fall from a height (24.0%). On arrival, 140 patients (20.9%) had a shock index of one or more, and 163 patients (24.3%) received an emergency bleeding control procedure, among whom 65 patients (9.7%) received bleeding control surgery and 133 patients (19.8%) received interventional radiology treatment. Furthermore, 174 patients (25.9%) underwent intracranial surgery.

### 3.2. Effect of Earlier Door-to-CT Time on Mortality

**Figure 2:**
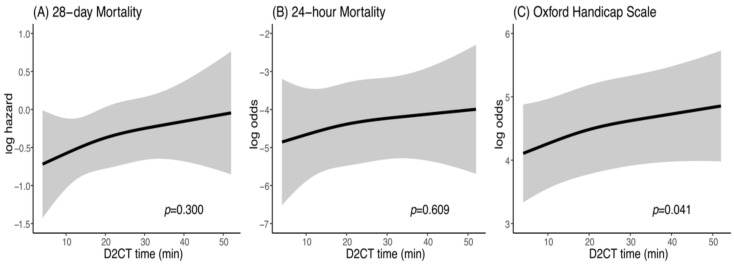
Effect of earlier D2CT on (**A**) 28-day mortality, (**B**) 24-h mortality and (**C**) Oxford Handicap Scale. PH, proportional hazard; CT, computed tomography; D2CT, door-to-CT.

A multivariable Cox proportional regression model was used to assess the associations between D2CT and 28-day mortality after adjusting for clinically important confounders. Table 2 provides the outcome data. The median D2CT was 19 (12 to 27) minutes. The rates of 28-day mortality and 24-h mortality and the median OHS were 16.7%, 9.7%, and 3 (2–5), respectively. In this analysis with a nonlinear cubic spline models, earlier D2CT was not significantly associated with either improved 28-day mortality (*p* = 0.30; Figure 2A) or with improved 24-h mortality (*p* = 0.609; Figure 2B). However, a beneficial association between D2CT and the OHS was observed (*p* = 0.041 (Figure 2C)).

### 3.3. Effect of Earlier Door-to-CT Time by Cause of Death

**Figure 3:**
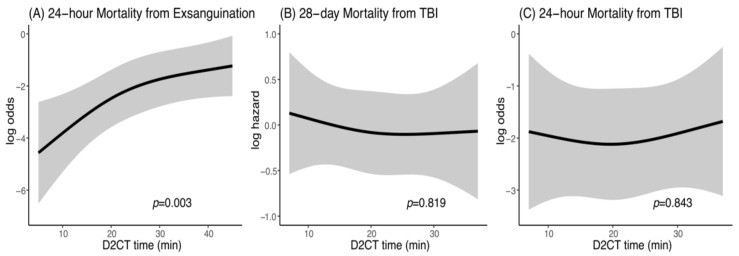
Effect of earlier D2CT on (**A**) 24-h mortality from exsanguination, (**B**) 28-day mortality from TBI, and (**C**) 24-h mortality from TBI. PH, proportional hazard; D2CT, door-to-CT; TBI, traumatic brain injury.

The effect of earlier D2CT on exsanguination and TBI was also evaluated in patients requiring a bleeding control procedure and in patients requiring intracranial surgery, respectively. The rates of 24-h mortality from exsanguination, 24-h mortality from TBI, and 28-day mortality from TBI were 4%, 5%, and 11%, respectively. In the patients requiring a bleeding control procedure, earlier D2CT was significantly associated with improved 24-h mortality from exsanguination (*p* = 0.003; Figure 3A). However, earlier D2CT was not associated with either 28-day mortality from TBI (*p* = 0.819; Figure 3B) or 24-h mortality (*p* = 0.843; Figure 3C) from TBI in the patients requiring intracranial surgery.

### 3.4. Effect of Earlier Door-to-Bleeding Control Time on Mortality

**Figure 4:**
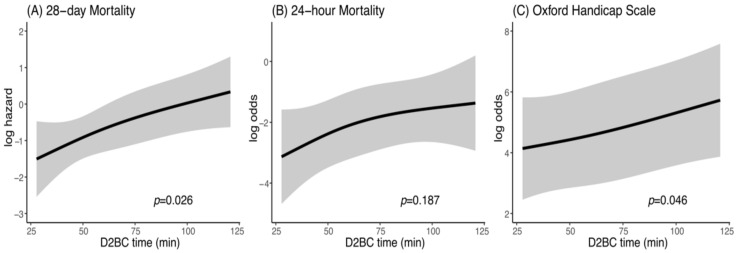
Effect of earlier D2BC on (**A**) 28-day mortality, (**B**) 24-h mortality, and (**C**) Oxford Handicap Scale. PH, proportional hazard; BC, bleeding control and D2BC, door-to-BC.

The effect of earlier D2BC on 28-day mortality was also assessed with a multivariable Cox proportional regression model. The median D2BC was 57 (45 to 75) minutes (Table 2). Earlier D2BC was significantly associated with improved 28-day mortality (*p* = 0.026; Figure 4A). In addition, the effects of D2BC on 24-h mortality and the OHS were evaluated. A beneficial trend was consistently observed for the assessment of earlier D2BC on 24-h mortality and the OHS (Figure 4B,C), although the association between D2BC and 24-h mortality was not statistically significant (*p* = 0.187).

## 4. Discussion

To our knowledge, this is the first study to examine the effect of earlier D2CT and D2BC on mortality in patients with severe blunt trauma. We developed a nonlinear cubic spline model in this analysis with adjustment for clinically important cofounders. We found that earlier D2CT was not associated with either improved all-cause 28-day mortality or 24-h mortality. However, if we evaluate the effect of D2CT by the cause of death, earlier D2CT was associated with decreased 24-h death from exsanguination, which is the main cause of preventable death. In terms of bleeding control, earlier D2BC was significantly associated with improved 28-day mortality, and this beneficial trend was consistently observed for the assessment of D2BC on secondary outcomes (24-h mortality and the OHS). In summary, earlier time to a hemostatic procedure such as bleeding control surgery and angioembolization was independently associated with decrease mortality. Meanwhile, time benefits of earlier CT examination were not observed for overall survival but were observed for decreased death from exsanguination in the patients requiring a hemostatic procedure.

The effect of time on patient outcomes has been well-reported, especially in the emergency field. For example, “door-to-balloon” time within 90 min is recommended as the standard of care for the treatment of ST elevation myocardial infarction under the principle that “time is muscle” [22]. Moreover, the concept of “time is brain” has been established to pursue the urgent management of patients with stroke [23]. As with other emergency fields, the results of the present study suggest that “time is blood” could be a standard for trauma management designed to shorten time to the control of life-threatening bleeding and improve mortality in patients with severe trauma.

Several studies have reported that minimizing time to laparotomy, and interventional radiology were associated with improved outcomes in trauma patients, likely by minimizing the time to hemostasis and the degree of blood loss [24,25,26]. A delay of more than 10 min to the operating room was independently associated with increased risk of mortality in hypotensive patients with gunshot wounds [24]. The probability of death increased by approximately 1% for each three min in patients with intra-abdominal injuries requiring laparotomy [25]. In the present study, earlier time to the start of the bleeding control procedure including operative management and interventional radiology showed a continuous association with better outcomes in severe blunt trauma.

In contrast, we found that there was no significant association between the time to CT examination and all-cause mortality. We included many patients without emergency procedures, possibly not the population to best benefit from early CT scanning, which might dilute the effect of earlier D2CT. In fact, if we look for an effect of early CT examination by cause of death, earlier D2CT was independently associated with decreased death from exsanguination. Our institution adapted trauma CT scan protocol, including arterial and venous phase for chest and abdominopelvic CT, which might contribute accurate diagnosis for vascular injuries and enabled timely bleeding control intervention [27,28]. Additionally, the prompt and accurate detection of traumatic injuries with CT scan would be helpful to distinguish between bleeding patients suitable for interventional radiology and surgical management. In the present study, earlier D2CT was not significantly associated with decreased death from TBI in patients requiring intracranial surgery. These findings indicate that earlier diagnostic examination would allow for more timely hemostatic intervention and contribute to better survival especially in patients requiring a definitive bleeding control procedure.

We address several limitations in this study. First, potential biases exist due to the retrospective study design. To cope with this limitation, we applied multivariable Cox proportional regression models adjusted for clinically important cofounders. Second, we included only patients who underwent CT examination, and thus, the results cannot be generalized to severely injured patients requiring surgical management in preference to CT scanning. Due to the exclusion of patients who received emergency surgery before CT examination, this study cannot address the superiority of direct operation without CT examination. Third, there could be several differences in the standard care because of the introduction of a CT scanner and interventional radiology system in our trauma resuscitation room midway through the study period, although the results reported in this study were consistent before and after the installation.

In conclusion, the earlier time for a hemostatic procedure was independently associated with decrease mortality. Meanwhile, time benefits of earlier CT examinations were not observed for the overall survival but were observed for decreased death from exsanguination in the patients requiring definitive bleeding control.

## Figures and Tables

**Table 1:** Baseline characteristics and diagnostic data of the study population.

Characteristics	*n* = 671
Age, years	51 (35–65)
Sex, male	463 (69.0%)
Mechanism of injury	
Motor vehicle accident	370 (55.1%)
Fall from a height	161 (24.0%)
Fall down steps	52 (7.7%)
Ground-level fall	32 (4.8%)
Crushed between objects	16 (2.4%)
Others	40 (6.0%)
GCS total score	13 (8–14)
HR, beats per min	91 (78–108)
Systolic BP, mm Hg	131 (78–108)
Shock index ≥ 1	140 (20.9%)
RR, per min	22 (18–28)
BT, °Celsius	36.5 (36.0–36.8)
RTS	7.1 (6.0–7.8)
Hb, g/dL	13.1 (11.8–14.4)
pH	7.39 (7.34–7.42)
Lactate, mmol/L	2.4 (1.6–3.6)
PT-INR	1.1 (1.1–1.2)
Activated partial thromboplastin time, s	29.9 (27.0–35.8)
AIS Head ≥ 3	477 (71.1%)
AIS Face ≥ 3	11 (1.6%)
AIS Chest ≥ 3	351 (52.3%)
AIS Abdomen ≥ 3	126 (18.7%)
AIS Extremities ≥ 3	232 (34.5%)
Injury Severity Score	26 (21–35)
Probability of survival	0.91 (0.71–0.97)
Emergency bleeding control procedure	163 (24.3%)
Bleeding control surgery	65 (9.7%)
Interventional radiology	133 (19.8%)
Intracranial surgery	174 (25.9%)

Categorical variables are presented as numbers (%), and continuous variables are presented as medians (25–75% percentile). GCS, Glasgow Coma Scale; HR, heart rate; BP, blood pressure; RR, respiratory rate; BT, body temperature; RTS, Revised Trauma Score; Hb, hemoglobin; PT-INR, prothrombin time-international normalized ratio and AIS, Abbreviated Injury Scale.

**Table 2:** Outcome data.

Parameter	Value
Door-to-CT time, min	19 (12–27)
Door-to-bleeding control time, min	57 (45–75)
28-day mortality	112 (17%)
Exsanguination	27 (4%)
TBI	73 (11%)
MODS	5 (1%)
Sepsis	2 (1%)
Respiratory	2 (1%)
Others	3 (1%)
24-h mortality	65 (10%)
Exsanguination	27 (4%)
TBI	36 (5%)
MODS	0 (0%)
Sepsis	0 (0%)
Respiratory	2 (1%)
Others	0 (0%)
Oxford Handicap Scale	3 (2–5)

Data are presented as numbers (%), and continuous variables are presented as medians (25–75% percentile). CT, computed tomography; TBI, traumatic brain injury and MODS, multiple organ dysfunction syndrome

## Data Availability

The data presented in this study are available on request from the corresponding author. The data are not publicly available due to their containing information that could compromise the privacy of research participants.

## Supplementary Materials

The following are available online at https://www.mdpi.com/article/10.3390/jcm10071522/s1: Table S1: Adjusted variables used for the assessment of D2CT and D2BC in the Cox proportional hazards regression model.
