# Supplementary material for: Effect of Earlier Door-to-CT and Door-to-Bleeding Control in Severe Blunt Trauma: A Retrospective Cohort Study
Source: J Clin Med. 2021 Apr 6;10(7):1522. doi: 10.3390/jcm10071522 (PMC8038745; doi:10.3390/jcm10071522)
Supplement: Supplementary file 1 [file jcm-10-01522-s001.pdf]

Supplementary Materials

Table S1. Adjusted variables used for the assessment of D2CT and D2BC in the Cox proportional hazards regression model.

Variables	D2CT	D2BC
Patient characteristics		
Age	✓	✓
Sex; Female/Male	✓	✓
Mechanism of injury		
Motor vehicle accident	✓	✓
Fall from a height	✓	✓
Others	✓	✓
Injury Severity Score	✓	✓
Revised Trauma Score	✓	✓
Deadly coagulopathy	✓	✓
Deadly acidosis	✓	✓
Deadly hyperthermia	✓	✓
Bleeding control procedure	✓	
Lactate	✓	✓
SOFA score	✓	✓
Heart rate	✓	✓
Body temperature	✓	✓
pH	✓	✓
Hb	✓	✓
PT-INR	✓	✓

D2CT, door-to-CT time; CT, computed tomography;D2BC, door-to-bleeding control; SOFA, Sequential Organ Failure Assessment; pH, potential of hydrogen; Hb, hemoglobin; PT-INR, prothrombin time-international normalized ratio.
